# Glycaemic Changes After Semaglutide Discontinuation in Adults With Type 1 Diabetes Using Automated Insulin Delivery: Analysis of the ADJUST‐T1D Extension Study

**DOI:** 10.1111/dom.71287

**Published:** 2026-08-31

**Authors:** Eslam Montaser, Kanchan Bhasin, Davida Kruger, Halis Kaan Akturk, Andrew Ahmann, Janet K. Snell‐Bergeon, Viral N. Shah

**Affiliations:** ^1^ Division of Endocrinology and Metabolism Indiana University School of Medicine Indianapolis Indiana USA; ^2^ Division of Endocrinology Diabetes and Metabolism Henry Ford Hospital Detroit Michigan USA; ^3^ Barbara Davis Center for Diabetes University of Colorado Aurora Colorado USA; ^4^ OHSU Harold Schnitzer Diabetes Health Center Portland Oregon USA; ^5^ Center for Diabetes and Metabolic Diseases Indiana University School of Medicine Indianapolis Indiana USA

**Keywords:** automated insulin delivery, continuous glucose monitoring, GLP‐1 receptor agonist, glycaemic variability, Type 1 diabetes

## Abstract

**Aims:**

To evaluate changes in glycaemic control following semaglutide discontinuation in adults with Type 1 diabetes and obesity who completed the ADJUST‐T1D randomised controlled trial.

**Materials and Methods:**

Continuous glucose monitoring (CGM) data were collected during a 12‐week extension phase after the ADJUST‐T1D trial. Analyses included participants previously randomised to semaglutide who discontinued treatment (*n* = 16) and participants previously randomised to placebo who did not receive a glucagon‐like peptide‐1 receptor agonist during follow‐up (*n* = 22). Changes in CGM‐derived metrics from Week 26 to the extension phase were compared using Wilcoxon rank‐sum tests with Benjamini–Hochberg adjustment. Analysis of covariance (ANCOVA) adjusting for Week 26 values was performed as a sensitivity analysis.

**Results:**

Compared with placebo, participants who discontinued semaglutide experienced a greater decline in time in range (70–180 mg/dL) (median change −3.6% vs. 1.5%; adjusted *p* = 0.044). Semaglutide discontinuation was also associated with greater increases in glucose standard deviation (6.0 vs. 1.2 mg/dL; adjusted *p* = 0.044) and coefficient of variation (3.1% vs. 1.1%; adjusted *p* = 0.044), indicating worsening glycaemic variability. Time above range (> 180 mg/dL) increased numerically but did not remain significant after adjustment (adjusted *p* = 0.064). No significant between‐group differences were observed in hypoglycaemia‐related CGM metrics. ANCOVA analyses showed consistent findings.

**Conclusions:**

In adults with Type 1 diabetes and obesity, semaglutide discontinuation was associated with a greater decline in time in range and increased glycaemic variability compared with placebo over 12 weeks. These findings suggest that glycaemic benefits achieved during treatment may not be fully sustained following withdrawal.

## 1 Introduction

ADJUST‐T1D, a multicentre, double‐blind, randomised controlled trial, demonstrated the superiority of semaglutide at doses of up to 1 mg/week compared with placebo (36% vs. 0%, *p* < 0.001) for the composite primary outcome in adults with Type 1 diabetes and obesity. The composite outcome was defined as meeting all three criteria: time in range (TIR; 70–180 mg/dL) greater than 70%, time below range (< 70 mg/dL) less than 4% and weight loss of at least 5% from baseline [1]. In addition, a post hoc analysis demonstrated a significant 30% reduction in insulin requirements in the semaglutide group compared with the placebo group [2].

While the benefits of glucagon‐like peptide‐1 receptor agonists (GLP‐1RAs) in glycaemic control are well‐established, studies in Type 2 diabetes suggest that their efficacy is not sustained upon discontinuation. For instance, a 4‐week off‐treatment follow‐up analysis of the SURPASS‐1 trial, discontinuation of tirzepatide was associated with a rapid increase in HbA1c of approximately 0.2% after treatment cessation, highlighting the potential for glycaemic deterioration following withdrawal of incretin‐based therapies [3]. A systematic review and meta‐analysis further corroborated this, reporting a significant metabolic rebound after GLP‐1RA discontinuation, characterised by an increase in HbA1c of 0.25% in individuals with obesity and 0.65% in those with Type 2 diabetes, alongside a deterioration in fasting plasma glucose [4]. Similarly, the STEP 1 extension trial, while primarily focusing on weight, observed that cardiometabolic improvements, including HbA1c, reverted towards baseline 1 year after semaglutide withdrawal [5]. In adults with Type 1 diabetes on pump therapy, semaglutide withdrawal has been shown to result in a rise in insulin requirements, predominantly from an increase in bolus insulin, and a numerical decrease in time‐in‐range [6].

The durability of glycaemic effects of semaglutide in adults with Type 1 diabetes after discontinuation remains a critical unknown. In this ADJUST T1D extension analysis, we assessed changes in continuous glucose monitoring (CGM)‐derived glycaemic metrics after discontinuation of semaglutide in adults with Type 1 diabetes using automated insulin delivery (AID) systems. We hypothesized that the glycaemic benefits observed during treatment would not be sustained following withdrawal, leading to a deterioration in glycaemic control. Semaglutide use in individuals with Type 1 diabetes may occur in different clinical contexts, including weight management in individuals with obesity and off‐label use for insulin resistance or excess weight in those without obesity. The metabolic consequences of treatment discontinuation may differ according to the indication for therapy, baseline metabolic characteristics and dosing regimen. Therefore, the present analysis specifically addresses adults with Type 1 diabetes and obesity treated with semaglutide in the context of obesity management, and the findings may not be directly generalisable to other populations receiving semaglutide.

## 2 Materials and Methods

### 2.1 Study Design and Population

This study is a post hoc extension analysis of ADJUST‐T1D. ADJUST‐T1D was a randomised, double‐blind, multicentre, parallel‐group, placebo‐controlled clinical trial evaluating semaglutide as an adjunct to AID in adults with Type 1 diabetes and obesity [1]. A total of 72 participants were randomised in a 1:1 ratio to receive either semaglutide (*n* = 36) or placebo (*n* = 36). Semaglutide was initiated at 0.25 mg once weekly for 4 weeks, followed by titration to 0.5 mg weekly and then 1 mg weekly through the 26‐week treatment period, with temporary dose reductions permitted in cases of intolerance.

After completion of the randomised trial, three academic centres participated in the extension phase. Approximately 3 months after each participant's final study visit, the clinical research coordinator at each centre reviewed the participant's electronic medical record (EMR). Both structured medication/prescription records and unstructured physician notes were reviewed to identify the use of any GLP‐1 receptor agonist therapy and to document the specific agent if prescribed. EMRs were also reviewed for the use of other glucose‐lowering medications; none of the participants were receiving additional glucose‐lowering therapies during the extension period. Participants who continued or initiated GLP‐1RA therapy were excluded from the present analysis. CGM data available in the EMR at the 3‐month extension time point were also collected. Participants were not contacted during the extension phase, and no prospective data on HbA1c or body weight were recorded because of the observational nature of the study. The study was approved by each centre's institutional review board.

Of 58 participants from three centres, 30 participants were originally randomised to the semaglutide group and 28 randomised to the placebo group. Participants were classified according to their original randomised assignment (prior semaglutide exposure versus placebo exposure). To specifically evaluate the effects associated with semaglutide therapy discontinuation, analyses were restricted to participants who did not receive any GLP‐1RA drug during the extension phase (either daily or weekly GLP‐1RA), and who had CGM data available for analysis (Figure 1).

**FIGURE 1:**
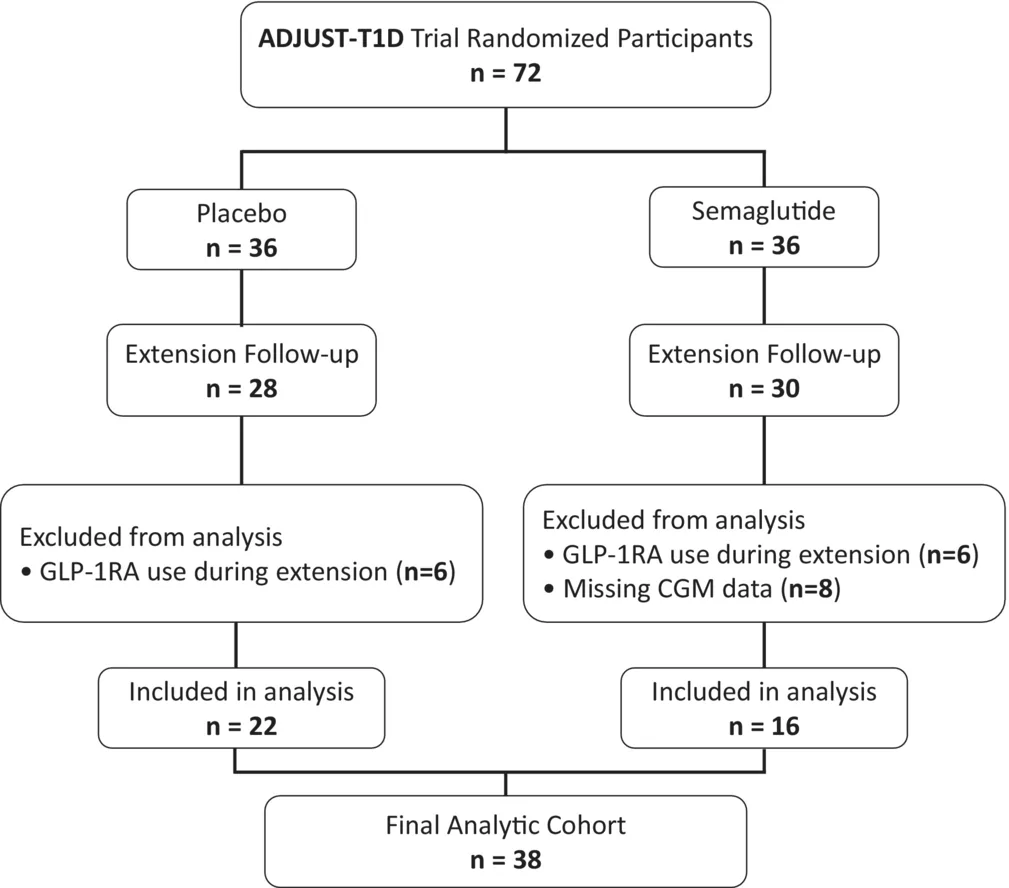
Schematic overview of study design, participant selection, exclusions, group allocation and the final analytical cohort for the 3‐month ADJUST‐T1D extension study. Participants who continued semaglutide therapy during the extension phase or had incomplete follow‐up CGM data were excluded from the analysis.

### 2.2 Continuous Glucose Monitoring Metrics

Up to 14 consecutive days of CGM data obtained at Visit 6 (Week 26) and at or prior to the 3‐month extension visit were used to estimate TIR (70–180 mg/dL), time in tight range (TITR; 70–140 mg/dL), time above range (TAR; > 180 mg/dL), time in severe hyperglycaemia (TAR > 250 mg/dL), time below range (TBR; < 70 mg/dL), time in clinically significant hypoglycaemia (TBR < 54 mg/dL), mean glucose, standard deviation (SD) and coefficient of variation (CV). To ensure high‐quality CGM data, the first 12 h following sensor insertion were excluded to minimise sensor stabilisation effects. Days containing more than 20% missing CGM data were excluded, and participants were required to have > 70% active CGM data. Missing intervals shorter than 60 min were linearly interpolated.

### 2.3 Statistical Analysis

CGM‐derived metrics at Week 26 (end of the randomised treatment period) were summarised as median (interquartile range [IQR]) or counts and percentages, as appropriate. Between‐group comparisons were performed using Wilcoxon rank‐sum tests for continuous variables and Fisher's exact test for categorical variables.

Changes in CGM‐derived metrics from Week 26 to the extension phase (12 weeks after completion of the randomised trial) were calculated for each participant as follows: Δ = extension phase − Week 26. Within each treatment group, changes are presented as median (IQR). Between‐group differences in changes from Week 26 to the extension phase were assessed using the Wilcoxon rank‐sum test. To account for multiple comparisons across CGM‐derived metrics, *p* values were adjusted using the Benjamini–Hochberg procedure.

To account for differences in glycaemic status at Week 26 and potential regression to the mean, analysis of covariance (ANCOVA) models were constructed for each CGM‐derived metric, with the extension‐phase value as the dependent variable, treatment group as the independent variable and the corresponding Week 26 value included as a covariate. The regression coefficient (*β*) for treatment group represents the adjusted mean difference between participants previously exposed to semaglutide and those assigned to placebo, after accounting for the corresponding Week 26 value. Standard errors (SEs) are reported for all model coefficients. Model assumptions were evaluated using standard diagnostic procedures. ANCOVA was selected because the extension analysis evaluated a single post‐discontinuation assessment, with the corresponding Week 26 value serving as the reference covariate, rather than multiple repeated longitudinal measurements that would require a mixed‐effects modelling approach.

All statistical tests were two‐sided. Statistical significance was assessed using adjusted *p* < 0.05. All analyses were performed using R version 4.0.5 (R Foundation for Statistical Computing, Vienna, Austria).

## 3 Results

### 3.1 Study Population

Of 72 participants randomised in the ADJUST T1D trial (36 placebo, 36 semaglutide), 58 participants were followed up to 3 months during the extension phase (28 placebo, 30 semaglutide). Participants who received a GLP‐1RA during the extension phase were excluded (placebo, *n* = 6; prior semaglutide, *n* = 6). In addition, eight participants in the prior semaglutide group were excluded because of missing CGM data. The final analytic sample included 38 participants: 22 in the placebo group and 16 in the prior semaglutide group (Figure 1).

### 3.2 Characteristics at the End of the Randomised Treatment Phase (Week 26)

Characteristics at the Week‐26 visit, which marked entry into the extension phase, are summarised in Table 1. At V6, participants in the prior semaglutide group exhibited more favourable metabolic profiles compared with the placebo group, including lower HbA1c (6.85% vs. 7.40%, *p* = 0.039), lower BMI (31.2 vs. 33.7 kg/m^2^, *p* = 0.049) and lower glycaemic variability as reflected by CV (33.15% vs. 37.17%, *p* = 0.020).

**TABLE 1:** Clinical characteristics and Week 26 continuous glucose monitoring (CGM) metrics of participants included in the ADJUST‐T1D extension analysis.

Characteristics	Placebo (*N* = 22)	Semaglutide (*N* = 16)	*p*
Age (years)	36 (25–54)	38 (36–51)	0.30
Sex—female (%)	12 (55%)	10 (63%)	0.70
HbA1c (%)	7.4 (7.1–7.6)	6.9 (6.5–7.5)	0.039
BMI (kg/m^2^)	33.7 (32.0–37.3)	31.2 (26.9–34.1)	0.049
CGM—metrics			
TIR (70–180 mg/dL)	61.5 (53.3–65.9)	61.8 (52.4–75.9)	0.60
TITR (70–140 mg/dL)	33.7 (29.6–41.4)	35.2 (25.9–44.9)	0.99
TAR180 (time above 180 mg/dL)	35.9 (32.1–46.5)	37.2 (23.0–47.3)	0.70
TBR70 (time below 70 mg/dL)	1.3 (0.4–2.9)	0.7 (0.3–1.2)	0.13
TAR250 (Time above 250 mg/dL)	11.8 (9.1–15.1)	11.5 (4.7–14.3)	0.60
TBR54 (Time below 54 mg/dL)	0.33 (0.02–0.6)	0.2 (0.03–0.2)	0.11
Mean glucose	169.9 (161.5–185.2)	171.4 (153.2–184.5)	0.70
SD	62.9 (54.7–71.2)	56.4 (48.5–61.6)	0.08
CV (%)	37.2 (34.3–41.0)	33.2 (30.1–35.5)	0.02

*Note:* Data are presented as median (interquartile range) or *n* (%). Age is reported at study enrolment (baseline—V0), whereas body mass index (BMI) and all CGM‐derived metrics were assessed at Week 26 (end of the randomised treatment period and baseline of the extension phase). Between‐group comparisons were performed using Wilcoxon rank‐sum tests for continuous variables and Fisher's exact test for categorical variables. CGM metrics included time in range (TIR; 70–180 mg/dL), time above range (TAR; > 180 mg/dL), time in severe hyperglycaemia (TAR > 250 mg/dL), time in tight range (TITR; 70–140 mg/dL), time below range (TBR; < 70 mg/dL and < 54 mg/dL), mean glucose, standard deviation (SD) and coefficient of variation (CV).

### 3.3 Changes in CGM‐Derived Metrics

Changes in CGM‐derived metrics from Week 26 (V6) to the extension phase are shown in Table 2. Compared with placebo, participants with prior semaglutide exposure experienced a greater decline in TIR (70–180 mg/dL) following treatment discontinuation (median change −3.6% vs. 1.5%; adjusted *p* = 0.044). Measures of glycaemic variability also worsened after semaglutide discontinuation, as reflected by greater increases in glucose SD (6.0 vs. 1.2 mg/dL; adjusted *p* = 0.044) and CV (3.1% vs. 1.1%; adjusted *p* = 0.044). A non‐significant increase in TAR (> 180 mg/dL) was observed in participants with prior semaglutide exposure (3.9% vs. −0.7%; adjusted *p* = 0.064). No significant between‐group differences were observed for mean glucose, time below range < 70 mg/dL or < 54 mg/dL, time above 250 mg/dL or TITR (70–140 mg/dL). Before adjustment for multiple comparisons, between‐group differences were nominally significant for TIR (*p* = 0.008), TAR > 180 mg/dL (*p* = 0.029), SD (*p* = 0.011) and CV (*p* = 0.015). Following Benjamini–Hochberg adjustment, TIR, SD and CV remained statistically significant, whereas TAR > 180 mg/dL did not. The longitudinal trajectory of TIR across baseline (V0), Week 26 (V6) and the extension visit is shown in Figure 2.

**TABLE 2:** Changes in continuous glucose monitoring–derived glycaemic metrics from end‐of‐treatment visit (V6—Week 26) to extension phase (post‐discontinuation follow‐up).

Metric	Placebo (*n* = 22) Δ (extension 3 months − Week 26)	Semaglutide (*n* = 16) Δ (extension 3 months − Week 26)	Adjusted *p*
ΔTIR (%)	1.5 (−3.1 to 3.9)	−3.6 (−9.8 to −0.6)	**0.044**
ΔTITR (%)	1.0 (−1.6 to 5.1)	−1.2 (−7.4 to 0.1)	0.108
ΔTAR > 180 (%)	−0.7 (−3.6 to 2.8)	3.9 (0.0 to 10.0)	0.064
ΔTAR > 250 (%)	−0.1 (−1.7 to 4.0)	2.4 (−0.1 to 6.2)	0.233
ΔMean glucose (mg/dL)	0.0 (−4.1 to 3.3)	4.5 (−2.7 to 14.5)	0.233
ΔSD (mg/dL)	1.2 (−2.9 to 5.0)	6.0 (1.7 to 11.3)	**0.044**
ΔCV (%)	1.1 (−2.7 to 2.4)	3.1 (0.3 to 5.0)	**0.044**
ΔTBR < 70 (%)	0.1 (−0.9 to 0.2)	0.2 (−0.3 to 1.2)	0.305
ΔTBR < 54 (%)	0.0 (−0.3 to 0.1)	0.1 (−0.0 to 0.2)	0.341

*Note:* Data are presented as median (interquartile range). Changes were calculated for each participant as extension‐phase values (post‐discontinuation follow‐up) minus Week 26 values (end‐of‐treatment visit). *p* Values were adjusted using the Benjamini–Hochberg procedure. CGM metrics included time in range (TIR; 70–180 mg/dL), time above range (TAR; > 180 mg/dL), time in severe hyperglycaemia (TAR > 250 mg/dL), time in tight range (TITR; 70–140 mg/dL), time below range (TBR; < 70 mg/dL and < 54 mg/dL), mean glucose, standard deviation (SD) and coefficient of variation (CV). Bold values indicate adjusted *p* < 0.05.

**FIGURE 2:**
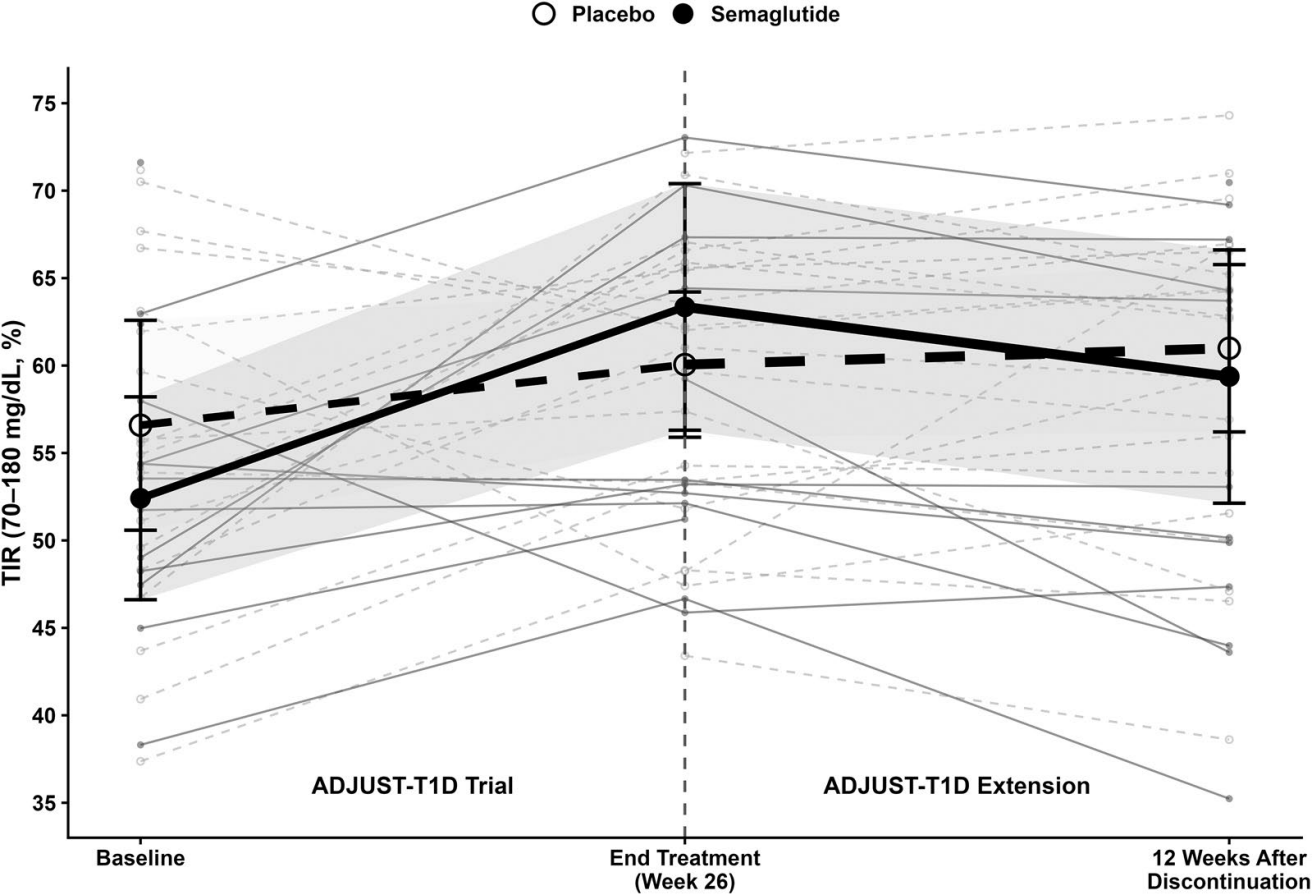
Longitudinal changes in time in range (TIR) following semaglutide discontinuation in adults with Type 1 diabetes. TIR (70–180 mg/dL) is shown at baseline, end of randomised treatment (Week 26) and 12 weeks after treatment discontinuation. Thin grey lines represent individual participant trajectories, with lighter dashed lines for placebo and darker solid lines for semaglutide. Thick black lines and larger points represent group mean trajectories, with open and filled circles indicating placebo and semaglutide groups, respectively. Error bars represent 95% confidence intervals, and shaded ribbons indicate the corresponding uncertainty around the mean trajectories. The vertical dashed line indicates the transition from the randomised ADJUST‐T1D trial to the extension phase.

In absolute terms, glycaemic variability at the extension visit was similar between groups, with median CV of 36.1% (IQR, 33.7–38.6) versus 36.6% (33.1–42.3) and median SD of 61.0 (56.0–64.5) versus 63.1 mg/dL (54.7–72.7) in the prior‐semaglutide and placebo groups, respectively. The greater increases in CV and SD therefore reflected a loss of the more favourable Week‐26 glycaemic variability profile in the prior‐semaglutide group rather than markedly higher absolute variability at the end of follow‐up.

### 3.4 Adjusted Analyses

As a sensitivity analysis, ANCOVA models were constructed to adjust for the corresponding Week‐26 CGM metric at the start of the extension phase. After adjustment for corresponding Week 26 values, participants with prior semaglutide exposure had lower TIR during the extension phase than participants previously assigned to placebo (*β* = −5.01, SE = 2.53, *p* = 0.054).

Prior semaglutide exposure was also associated with higher TAR (> 180 mg/dL) (*β* = 4.59, SE = 2.64, *p* = 0.089) and higher CV (*β* = 2.02, SE = 1.09, *p* = 0.071), although these associations did not reach statistical significance. In contrast, after adjustment for week‐26 SD, prior semaglutide exposure remained significantly associated with higher SD during the extension phase (*β* = 6.73, SE = 2.60, *p* = 0.013).

Week‐26 CGM metrics at extension entry were strongly associated with extension‐phase outcomes across models (e.g., TIR: *β* = 0.85, *p* < 0.001), and the TIR model explained a substantial proportion of outcome variability (*R* ^2^ = 0.57). Although these adjusted associations did not reach conventional statistical significance, the direction and magnitude of the effects were consistent with the primary analyses, suggesting that glycaemic benefits observed during semaglutide treatment may not be fully sustained following treatment discontinuation.

## 4 Discussion

In this extension analysis of the ADJUST‐T1D randomised controlled trial, discontinuation of semaglutide for 12 weeks was associated with a greater decline in TIR and increased glycaemic variability among adults with Type 1 diabetes and obesity using AID systems. Compared with participants previously assigned to placebo, those with prior semaglutide exposure experienced a greater reduction in TIR and greater increases in glucose SD and CV during the extension phase. TAR also increased numerically after semaglutide discontinuation but did not reach statistical significance. These findings suggest that the glycaemic benefits achieved during semaglutide treatment may not be fully sustained following treatment withdrawal, even in individuals using AID systems.

The observed ‘metabolic rebound’ in our Type 1 diabetes cohort aligns with and extends findings previously reported in Type 2 diabetes and obesity populations. The SURPASS‐1 and STEP 1 extension trials demonstrated that glycaemic benefits, including HbA1c improvement, and broader cardiometabolic benefits tend to revert towards baseline following withdrawal of tirzepatide or semaglutide [3, 5]. Our findings extend these observations by demonstrating that changes following GLP‐1 receptor agonist withdrawal can also be detected using CGM‐derived measures in adults with Type 1 diabetes receiving AID therapy. Notably, the rapid rise in HbA1c (0.2%–0.3% within 4 weeks) reported after tirzepatide withdrawal in Type 2 diabetes [3], together with broader metabolic deterioration identified in recent meta‐analyses [4, 7, 8], suggests that treatment withdrawal may result in reversal of treatment‐associated metabolic benefits across different diabetes populations. The use of CGM in the present study provides a more granular assessment of this process, identifying changes in TIR and glycaemic variability that may not be fully captured by HbA1c alone.

Differences in lifestyle intervention should also be considered when comparing the present findings with studies in obesity and Type 2 diabetes. The ADJUST‐T1D trial did not include a structured lifestyle‐counselling intervention, whereas lifestyle modification was incorporated into the management strategy in some previous obesity and Type 2 diabetes trials. Differences in lifestyle support may influence both the magnitude of treatment‐associated improvements and the trajectory following medication withdrawal. Thus, the magnitude of glycaemic ‘reversion’ observed in the present study may not be directly comparable with that reported in trials with more intensive lifestyle intervention. However, lifestyle behaviours were not systematically assessed during the extension phase, and therefore their independent contribution to the observed changes cannot be determined.

Because the objective of this study was to evaluate glycaemic changes following treatment withdrawal, Week 26 served as the clinically relevant reference point for the adjusted analyses, representing glycaemic status immediately before semaglutide discontinuation. In sensitivity analyses adjusting for the corresponding Week‐26 CGM values, the direction of the associations remained consistent, although the differences in TIR, TAR and CV did not reach statistical significance. In contrast, the association between prior semaglutide exposure and increased SD remained significant after adjustment. These findings suggest that the observed between‐group differences should be interpreted cautiously, particularly for TIR, while the persistence of the SD association supports a potential effect of treatment discontinuation on glycaemic variability.

From a mechanistic perspective, these findings are consistent with the reversible nature of GLP‐1 receptor agonist effects. Semaglutide enhances glucose‐dependent insulin secretion, suppresses glucagon release, delays gastric emptying and reduces appetite. Withdrawal of these effects may allow pre‐treatment physiological patterns to re‐emerge, potentially contributing to increased glycaemic variability and reduced postprandial control. Changes in appetite, food intake and gastric emptying may be particularly relevant following treatment discontinuation. Semaglutide reduces appetite through central mechanisms and delays gastric emptying; loss of these effects may contribute to increased energy intake and greater postprandial glucose excursions. However, appetite, dietary intake and gastric emptying were not directly assessed during the extension phase, and their contribution to the observed glycaemic changes cannot be determined. These findings are consistent with clinical observations that GLP‐1 receptor agonists function as chronic disease‐modifying therapies rather than curative interventions [9, 10, 11].

A key strength of the present study is the use of CGM‐derived metrics, which provided information beyond conventional measures of glycaemic control. In particular, glycaemic variability, assessed using SD and CV, increased following semaglutide discontinuation. Glycaemic variability has been increasingly recognised as an important component of glycaemic control and may provide information not fully captured by HbA1c alone [12, 13, 14]. The persistence of the association between prior semaglutide exposure and higher SD after adjustment for Week‐26 values suggests that glycaemic variability may be a sensitive marker of the loss of treatment‐associated benefit following discontinuation.

Several limitations should be considered. First, this was an exploratory post hoc analysis with a relatively small sample size, and the extension phase was observational. Second, differential attrition may have influenced the findings. Eight participants originally assigned to semaglutide were excluded because CGM data were unavailable, whereas no participants included in the placebo analysis were excluded for missing CGM data. Available Week‐26 clinical characteristics were comparable between included and excluded participants, with no significant differences in age, sex distribution, HbA1c or BMI (Table S1). Nevertheless, CGM‐derived measures were unavailable for excluded participants, and informative missingness cannot therefore be completely excluded. In addition, participants who continued GLP‐1 receptor agonist therapy during the extension were excluded from the discontinuation analysis, which may also have introduced selection bias.

Third, although CGM provided a detailed assessment of glycaemic control, HbA1c, body weight and insulin dose changes were not the primary focus of this extension analysis. Comprehensive longitudinal insulin‐dose data were not systematically available for all participants, limiting our ability to evaluate changes in insulin requirements following semaglutide discontinuation. Fourth, participants previously assigned to semaglutide had a more favourable glycaemic profile at Week 26 for several measures, raising the possibility of regression to the mean. Adjustment for corresponding Week‐26 CGM values attenuated some between‐group differences, although the overall direction of the findings remained consistent. Regression to the mean may therefore have contributed to some of the observed changes, particularly for measures of glycaemic variability. The persistence of the association between prior semaglutide exposure and higher SD after adjustment, however, supports a potential association between discontinuation and worsening glycaemic variability.

From a clinical perspective, these findings suggest that the glycaemic benefits associated with semaglutide may require continued treatment to be maintained, even among adults with Type 1 diabetes using AID systems. The findings also highlight the potential importance of considering a structured transition strategy when GLP‐1 receptor agonist therapy is discontinued. This is consistent with broader evidence indicating that discontinuation of GLP‐1 receptor agonists is frequently associated with loss of therapeutic benefits [4, 15]. However, the present findings should not be interpreted as establishing that semaglutide discontinuation inevitably results in clinically important deterioration in all glycaemic outcomes, particularly given the relatively small sample size and the lack of statistically significant differences in several adjusted analyses.

Future studies should evaluate whether the trajectory following semaglutide discontinuation differs according to residual endogenous insulin secretion, including in individuals with and without detectable C‐peptide. Larger prospective studies with longer follow‐up should also assess the broader cardiometabolic consequences of discontinuation, including changes in insulin resistance, lipid profiles, blood pressure, waist circumference, body weight and concomitant medication use. Systematic assessment of insulin requirements, dietary intake, appetite and other behavioural factors would help clarify the mechanisms underlying glycaemic changes after treatment withdrawal. Such studies may also help determine whether step‐down approaches, combination therapies or behavioural interventions can mitigate the loss of treatment‐associated benefits.

In conclusion, among adults with Type 1 diabetes and obesity using AID systems, discontinuation of semaglutide was associated with a greater decline in TIR and increased glycaemic variability compared with placebo. These findings suggest that the glycaemic benefits achieved during semaglutide treatment may not be fully sustained following treatment withdrawal and support the need for larger prospective studies to better define the durability and clinical consequences of GLP‐1 receptor agonist discontinuation in Type 1 diabetes.

## Author Contributions

**Eslam Montaser:** conceptualisation, data curation, formal analysis, methodology, software, visualisation, writing – original draft, writing – review and editing. **Kanchan Bhasin:** investigation, data curation, writing – review and editing. **Davida Kruger:** conceptualisation, investigation, writing – review and editing. **Halis Kaan Akturk:** conceptualisation, investigation, writing – review and editing. **Andrew Ahmann:** conceptualisation, investigation, writing – review and editing. **Janet K. Snell‐Bergeon:** conceptualisation, investigation, writing – review and editing. **Viral N. Shah:** conceptualisation, funding acquisition, investigation, project administration, resources, supervision, writing – review and editing. All authors contributed to the revision of the article and approved the final version.

## Funding

This study was supported by Breakthrough T1D (formerly JDRF). Novo Nordisk provided the study drug free of charge. The opinions expressed in this manuscript are solely those of the author(s) and do not necessarily represent the views of Novo Nordisk.

## Conflicts of Interest

D.K. has received speaker honoraria from Abbott, Dexcom, Novo Nordisk, Eli Lilly, Corcept, CeQur, Insulet, Tandem Diabetes Care and Sequel; has served on advisory boards for Abbott, Dexcom, Eli Lilly, Novo Nordisk, Corcept, Insulet, MannKind, MiniMed and Sequel and has received research support from Abbott, Insulet, Sequel, Novo Nordisk and Tandem Diabetes Care. H.K.A. has received honoraria and research support through the University of Colorado from Dexcom, Abbott Diabetes Care, Roche, MiniMed and MannKind. A.A. has received honoraria from Medtronic for participation on an advisory board. J.K.S.‐B. has nothing to disclose. V.N.S.'s institution has received research support from Dexcom, Insulet, MiniMed, Eli Lilly, the Cystic Fibrosis Foundation, Zucara Therapeutics, Enable Bioscience, DEKA Research, the National Institutes of Health (NIH) and Breakthrough T1D. Abbott and Dexcom provided CGM sensors at no cost for investigator‐initiated studies, and Novo Nordisk supplied the study drug at no cost for the ADJUST‐T1D trial. V.N.S. has received honoraria from Sanofi, Novo Nordisk, Eli Lilly, Roche, Dexcom, Insulet, Tandem Diabetes Care, MiniMed, Abbott Diabetes Care, Embecta and T1D Scout for consulting, advisory activities or speaking engagements outside the submitted work. The other authors declare no conflicts of interest.

## Supporting information


**Table S1:** Comparison of participants included and excluded from the extension analysis.

## Data Availability

The data analysed or generated during the study are available from the authors upon reasonable request.
