# Supplementary material for: Glycaemic Changes After Semaglutide Discontinuation in Adults With Type 1 Diabetes Using Automated Insulin Delivery: Analysis of the ADJUST‐T1D Extension Study
Source: Diabetes Obes Metab. 2026 Aug 31;28(11):10736–42. doi: 10.1111/dom.71287 (PMC13646344; doi:10.1111/dom.71287)
Supplement: Supplementary file 1 [file DOM-28-10736-s001.docx]

**Supplementary Table S1**. Comparison of participants included and excluded from the extension analysis.

| Prior randomised group | Analysis status | n | Age (years) | Female, n (%) | HbA1c (%) | BMI (kg/m²) |
| --- | --- | --- | --- | --- | --- | --- |
| Semaglutide | Included | 16 | 42.0 ± 11.1 | 10 (62.5) | 6.99 ± 0.68 | 31.6 ± 5.5 |
|  | Excluded – missing CGM | 8 | 36.0 ± 11.4 | 3 (37.5) | 7.29 ± 0.96 | 29.5 ± 2.0 |
|  | Excluded – continued semaglutide | 6 | 44.8 ± 11.7 | 4 (66.7) | 7.17 ± 0.57 | 31.6 ± 4.2 |
| Placebo | Included | 22 | 38.6 ± 14.1 | 11 (50.0) | 7.42 ± 0.54 | 35.0 ± 4.9 |
|  | Excluded – initiated semaglutide | 6 | 42.7 ± 11.9 | 3 (50.0) | 7.07 ± 0.30 | 37.3 ± 7.2 |

Data are presented as mean±SD. P-values comparing included participants with each excluded subgroup were not statistically significant for age, sex, HbA1c, or BMI (all p > 0.05).
